# Efficacy of Exenatide Administered Twice Daily in Body Mass Index Reduction in Patients with Type 2 Diabetes Mellitus

**DOI:** 10.1155/2022/7128859

**Published:** 2022-10-10

**Authors:** Jie Zhang, Tong-Zhang Xian, Yu Teng, Xiuzhi Wang, Ming-Xiao Wu, Chen Li, Weihao Wang, Fuli Man, Xianbo Zhang, Xiaoxia Wang, Li-Xin Guo

**Affiliations:** ^1^Department of Endocrinology, Beijing Hospital, National Center of Gerontology, Institute of Geriatric Medicine, Chinese Academy of Medical Sciences, Graduate School of Peking Union Medical College, Beijing 100730, China; ^2^Central Laboratory, Beijing Obstetrics and Gynecology Hospital, Capital Medical University, Beijing, China; ^3^Department of Endocrinology, Pinggu Hospital, Beijing Traditional Chinese Medicine Hospital, Beijing 100006, China; ^4^Department of Ultrasound, Beijing Hospital, National Center of Gerontology, Institute of Geriatric Medicine, Chinese Academy of Medical Sciences, Beijing 100730, China

## Abstract

**Background:**

Exenatide is a glucagon-like peptide-1 receptor agonist that can reduce body weight. This study aimed to determine the efficacy of exenatide on body mass index (BMI) reduction in patients with type 2 diabetes mellitus (T2DM) with differing baseline body weight, blood glucose, and atherosclerotic status and to determine if there is a correlation between BMI reduction and cardiometabolic indices in these patients.

**Methods:**

This retrospective cohort study used data from our randomized controlled trial. A total of 27 T2DM patients treated with combination therapy of exenatide twice daily and metformin for 52 weeks were included. The primary endpoint was a change in the BMI from the baseline to week 52. The secondary endpoint was a correlation between BMI reduction and cardiometabolic indices. *Findings*. The BMIs of overweight and obesity patients and those with glycated hemoglobin (HbA1c) ≥ 9% significantly decreased −1.42 ± 1.48 kg/m^2^(*P*=0.015) and −0.87 ± 0.93 kg/m^2^(*P*=0.003), respectively, at the baseline after 52 weeks of treatment. There was no reduction in BMI in patients with normal weight, HbA1c <9%, the nonatherosclerosis group, and the atherosclerosis group. The decrease in BMI was positively correlated with changes in blood glucose, high-sensitivity C-reactive protein (hsCRP), and systolic blood pressure (SBP).

**Conclusion:**

BMI scores improved after exenatide treatment for 52 weeks in T2DM patients. Weight loss was affected by baseline body weight and blood glucose level. In addition, BMI reduction from the baseline to 52 weeks was positively correlated with baseline HbA1c, hsCRP, and SBP. Trial Registration. Chinese Clinical Trial Registry (ChiCTR-1800015658).

## 1. Introduction

Obesity is characterized by excessive accumulation of visceral and subcutaneous fat and is the common concomitant condition of type 2 diabetes mellitus (T2DM) [1]. Approximately 60% of T2DM patients in China are overweight and obese [2]. Weight gain is a major risk factor for T2DM as it induces chronic inflammation, endoplasmic reticulum stress, mitochondrial dysfunction, and insulin resistance [1, 3]. Abdominal obesity is also an important factor for arteriosclerosis [4]. Obesity promotes atherosclerosis by activating migration of endothelial cells, increasing smooth muscle cell proliferation, and promoting vascular calcification [5] In addition, fibrinogen, platelet count, blood lipids, and plasma viscosity are significantly higher in obese individuals than in normal-weight people [5] Deformation of red blood cells also decreases with obesity, resulting in abnormal hemorheology, circulatory dysfunction, tissue blood perfusion disorders, and increased risk of thrombosis and arteriovenous atherosclerosis formation [6]. Sustained moderate weight loss of more than 5% of initial body weight provides significant benefits for blood glucose, blood pressure, blood lipid, and other metabolic indices in patients with T2DM [7–9]. Therefore, weight reduction has been considered a key element of patient-centered approaches for the management of T2DM [10, 11].

Glucagon-like peptide-1 receptor agonists (GLP-1RAs) have a variety of glucose regulation effects, including enhancing glucose-dependent insulin secretion, reducing glucagon secretion, reducing food intake, and slowing gastric emptying [12–14]. In addition to lowering the incidence of hypoglycemia, GLP-1RAs can reduce body weight [13–18]. Recent guidelines [19–21] have recommended that GLP-1RAs be considered for individuals with T2DM who require weight loss regardless of glycated hemoglobin (HbA1c) levels. Exenatide is a GLP-1RA that has been shown to be effective for reducing body weight and the body mass index (BMI) [22, 23]. One study showed that treatment with exenatide for 48 weeks decreased body weight in normal-weight, overweight, and obese patients by 3.7% (–2.2 ± 2.8 kg; *P* < 0.001), 5.5% (–3.9 ± 3.2 kg; *P* < 0.001), and 5.7% (–4.0 ± 5.4 kg; *P* < 0.01) at baseline levels, respectively [24].

A previous study also suggested that the baseline BMI was closely related to weight change and glycemic control after exenatide monotherapy [24]. However, the effect of GLP-1RAs on body weight reduction in patients with T2DM with differing baseline BMI measurements has not been extensively studied [24]. Furthermore, little is known about the influence of baseline blood glucose and the presence of atherosclerosis on weight loss efficacy after GLP-1RA treatment in T2DM patients. The purpose of this retrospective cohort study was to compare weight loss efficacy of exenatide in T2DM patients with differing baseline BMI, blood glucose, and atherosclerotic levels and to determine if there is a correlation between BMI reduction and cardiometabolic indices in order to identify patient characteristics that could result in good response to exenatide.

## 2. Methods

### 2.1. Patients

This was a retrospective cohort study using data from our randomized clinical trial that investigated the effects of exenatide, administered twice daily, combined with metformin when compared to insulin on carotid intima-media thickness (cIMT) in patients with T2DM [23]. The original study was performed in accordance with the Declaration of Helsinki, and the study protocol was reviewed and approved by the Research Ethics Committee of Beijing Hospital (No. 2013 BJYYEC-017A-03). The study was registered in the Chinese Clinical Trial Registry (ChiCTR-1800015658). After all participants were informed of the study details, they signed the corresponding consent forms.

### 2.2. Patient Subgroups

A total of 27 patients who were treated with exenatide twice daily and metformin for 52 weeks were included in this analysis. According to baseline levels of body weight, glycemic profile, and atherosclerotic status, the patients were classified into the following subgroups: BMI <24 kg/m^2^ (normal-weight); BMI ≥24 kg/m^2^ (overweight and obese); HbA1c < 9% (good glycemic control); HbA1c ≥ 9% (poor glycemic control); cIMT <1 mm (mild atherosclerosis); cIMT ≥1 mm (severe atherosclerosis).

### 2.3. Medications

Exenatide injection (BYETTA®): 5 ug per dose, 60 doses, 1.2 mL prefilled pen; 10 ug per dose, 60 doses, 2.4 mL prefilled pen, AstraZeneca, Baxter Pharmaceutical Solutions LLC., Indiana, USA.

Metformin hydrochloride tablets (Glucophage): 500 mg per tablet, Sino-American Shanghai Squibb Pharmaceuticals Ltd, China.

Exenatide was administered 5 *μ*g twice daily subcutaneously, 60 min before breakfast and dinner. The exenatide dose was titrated to 10 *μ*g twice daily after 4 weeks. Metformin 500 mg was administered three times daily according to the blood glucose level.

### 2.4. Outcomes

The primary endpoint was a change in the BMI from the baseline to week 52. The secondary endpoints were correlations between BMI reduction and cardiometabolic indices: HbA1c, high-sensitivity C-reactive protein (hsCRP), systolic blood pressure (SBP), and low-density lipoprotein cholesterol (LDL-C).

### 2.5. Statistical Analysis

Data were analyzed using SPSS statistical software version 22 for Windows (IBM Corp., Armonk, NY, USA). Data are expressed as means and standard deviations (SD). The normality of the data was analyzed using the Kolmogorov–Smirnov test. The paired *t*-test or the Wilcoxon signed-rank test was used for within-group comparisons. Multiple linear regression was used to analyze correlations between weight, hsCRP, SBP, and LDL-C. A *P* value <0.05 indicated statistical significance.

## 3. Results

**Figure 1:**
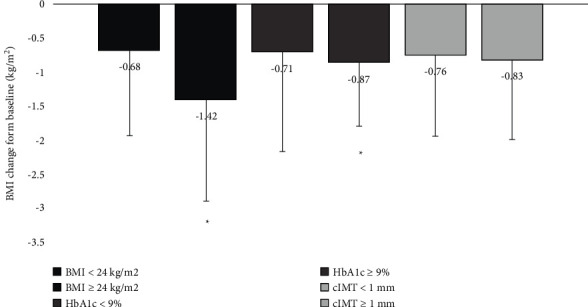
BMI reduction in T2DM patients with differing baseline characteristics after exenatide treatment for 52 weeks (^*∗*^*P* < 0.05*vs*. baseline).

The baseline characteristics of patients are shown in Table 1. After exenatide treatment, body weight decreased by −2.21 ± 3.43 kg at week 52 (*P*=0.005).  Figure 1 describes the efficacy of exenatide treatment in weight reduction in patients with differing baseline levels. A significant decrease in the BMI was observed in patients who had a BMI ≥24 kg/m^2^(*P*=0.015) and HbA1c ≥ 9% (*P*=0.003). There were no significant differences in BMI reduction between groups with differing baseline BMI (*P*=0.51), HbA1c (*P*=0.73), or cIMT (*P*=0.98) (Supplemental Figures 1–3).

Correlation analysis showed that decreases in the BMI were positively correlated with changes in HbA1c (*r* = 0.419, *P*=0.030), hsCRP (*r* = 0.440, *P*=0.022), and SBP (*r* = 0.510, *P*=0.007) at 52 weeks (Table 2).

## 4. Discussion

This retrospective cohort study showed that exenatide administered twice daily was clinically sufficient to significantly reduce body weight in T2DM patients with varying baseline levels of the BMI, suggesting a correlation between weight loss efficacy of exenatide and obesity.

The BMI of patients in all subgroups decreased at the baseline after 52 weeks of exenatide treatment, and this result was affected by obesity (baseline BMI). The statistically significant decrease in the BMI was found only in patients with a BMI ≥24 kg/m^2^ but not those with <24 kg/m^2^. A previous study found that exenatide reduced body weight in T2DM patients with different levels of obesity [25]. Another study observed the benefits of exenatide in the second week of treatment [26, 27]. A meta-analysis [28] of Asians also demonstrated that GLP-1RAs exhibited more benefits in T2DM patients with an overweight/obese BMI (*n* = 14; −0.44 kg; 95% confidence interval (CI) −0.62, −0.26; *I*^2^ = 85.3%) than in people with a normal BMI (*n* = 6; −0.03 kg; 95% CI −0.38, 0.32; *I*^2^ = 87.6%). However, body weight loss (*P*=0.572) was similar between the two groups. The beneficial effects of exenatide twice daily on body weight improvement were similar to those of exenatide once weekly [29] and liraglutide [30, 31].

We also found that weight loss efficacy of exenatide was influenced by hyperglycemic stages (baseline HbA1c). The decrease in the BMI after exenatide treatment tended to be slightly higher in patients with high baseline HbA1c values (HbA1c ≥ 9%) than those with low values (HbA1c < 9%); however, there was no significant difference between the groups. This finding suggests that exenatide could exhibit weight-lowering benefits for patients regardless of the baseline HbA1c level. The correlation analysis also revealed that BMI reduction after 52 weeks was positively correlated with HbA1c levels (*r* = 0.419, (*P*=0.030)). However, a prior study did not observe a correlation between weight loss and improvement in glycemic control [32]. Although 63.3% of the eligible participants who were treated with GLP-1RAs obtained dual benefits, including HbA1c reduction and weight loss, 3.8% of the patients did not respond to exenatide. Interestingly, 22% of the patients lost weight but had no improvement in glycemic control. In contrast, 11% of the patients gained weight but had blood glucose reduction. This is consistent with the result of another study of Korean obese T2DM patients that reported that the weight reduction effect of exenatide could not predict a decrease in HbA1c [33].

The magnitude of BMI reduction in patients with a BMI <24 kg/m^2^ and HbA1c < 9% was clinically significant, although there was lack of statistical significance. This might be related to the respective lifestyles of patients. Patients with normal weight tended to have healthier lifestyles and less weight changes than obese persons. Moreover, the average BMI value in the BMI <24 kg/m^2^ group was in a normal range at the baseline, which may explain the lack of a statistically significant effect of exenatide on the normal BMI group. Patients with better blood glucose control tended to have more strict dietary control, resulting in a weakening effect of GLP-1. Thus, the weight improvement was not statistically significant in these patients. Our multivariate linear regression model showed that weight loss from the baseline to 12 months was positively correlated with a patient's baseline weight and LDL-C and triglycerides levels and negatively correlated with prior insulin treatment [31]. Moreover, it has been documented that exenatide treatment twice daily was effective at lowering body weight in T2DM patients regardless of age, sex, race, and duration of diabetes [34, 35].

Obesity is a leading cause of cardiometabolic diseases [36]. Abnormal lipid metabolism, insulin resistance, inflammation, endothelial dysfunction, imbalance of adipokines, and activation of inflammatory bodies are considered the basis of atherosclerosis development in obese populations. Evidence showed that exenatide improved arteriosclerosis by reducing body weight, lowering LDL-C, and improving inflammatory responses [23]. In this study, there was no significant difference in weight reduction among T2DM patients who had differing baseline atherosclerotic statuses, suggesting that the presence of atherosclerosis did not affect the weight loss benefit of exenatide. Correlation analysis further revealed that BMI reduction after 52 weeks was positively correlated with hsCRP levels (*r* = 0.440, *P*=0.022), indicating that exenatide has potential for improving metabolic indices among T2DM patients. In our previous study, exenatide also significantly improved inflammation indices (CRP, fibrinogen, 8-hydroxydeoxyguanosine, irisin, and brain natriuretic peptide) from baseline levels [23].

There are some limitations in our study that should be addressed. Patients who received metformin alone were not included in the control arm of the analysis. The effects of exenatide treatment on clinical outcomes, especially BMI reduction, should be further analyzed in comparison with placebo as well as active comparators in future studies.

## 5. Conclusion

We found that exenatide has a significant effect on weight management in T2DM patients. Weight loss can be affected by baseline body weight and blood glucose level, and the benefits can be obtained regardless of arteriosclerosis status at the baseline.

Our study indicates that overweight or obese T2DM patients could respond better to exenatide than normal-weight patients without an increased risk of excess weight loss. Patients with poor blood glucose control can also benefit more from the weight loss effect of exenatide than patients with good blood glucose control. Patients with severe atherosclerosis can achieve the same weight loss effect as patients with mild atherosclerosis. In addition, the BMI reduction from the baseline to 52 weeks was positively correlated with baseline HbA1c, hsCRP, and SBP.

## Figures and Tables

**Table 1:** Patient demographics and baseline characteristics.

Variables	Normal-weight patients (*N* = 17)	Overweight and obese patients (*N* = 10)	*P* value
Age, y	60.47 ± 10.88	56.1 ± 15.21	0.392
HbA1c, %	8.54 ± 1.03	8.89 ± 1.07	0.413
FPG, mmol/L	9.92 ± 2.92	11.17 ± 2.99	0.301
eGFR, ml/min/1.73 m^2^	100.9 ± 12.32	105.8 ± 14.18	0.372
BMI, kg/m^2^	21.82 ± 1.27	26.75 ± 1.97	<0.0001
FIB, g/L	2.89 ± 0.68	2.88 ± 0.64	0.963
hs-CRP, mg/L	1.83 ± 1.89	4.79 ± 3.78	0.012
Irisin, pg/mL	71.04 ± 5.9	73.75 ± 4.66	0.252
8-oxo-gsn, ng/mL	6.58 ± 0.55	6.65 ± 0.42	0.701
NT-proBNP, pg/mL	220.2 ± 25.5	202.9 ± 19.18	0.111

Data are reported as the mean ± SD. *P* value was analyzed by the mean change comparison between normal-weight patients and overweight and obese patients. HbA1c: glycated hemoglobin; FPG: fasting plasma glucose; eGFR: estimated glomerular filtration rate; BMI: body mass index; NT-proBNP: N-terminal probrain natriuretic peptide.

**Table 2:** Correlations between changes in the BMI from baseline to week 52 and cardiometabolic indices after exenatide treatment.

	HbA1c	hsCRP	SBP	LDL-C
Correlation coefficient (*r*)	0.419	0.440	0.510	−0.270
*P* value	0.030	0.022	0.007	0.173

*P* value was analyzed by the mean change comparison between exenatide and insulin groups. HbA1c: glycated hemoglobin; hsCRP: high sensitivity C-reactive protein; SBP: systolic blood pressure; LDL-C: low-density lipoprotein cholesterol.

## Data Availability

The datasets generated and analyzed during the current study are available from the corresponding author on reasonable request.

## Additional Points

*What is already known about this subject?*: (i) Obesity is a common concomitant condition of T2DM. (ii) Sustained moderate weight loss of more than 5% of initial body weight provides significant benefits for blood glucose, blood pressure, blood lipid, and other metabolic indices in patients with T2DM. (iii) Glucagon-like peptide-1 receptor agonists (GLP-1RAs) have been considered for individuals with T2DM who require weight loss regardless of HbA1c levels. (iv) The baseline BMI affects weight change and glycemic control after exenatide monotherapy. *What are the new findings?*: (i) Exenatide significantly decreased BMI measurements in overweight and obese patients and in those with glycated hemoglobin (HbA1c) ≥ 9% after 52 weeks of treatment. (ii) Exenatide treatment did not reduce BMI measurements in healthy patients. (iii) BMI reduction after exenatide treatment for 52 weeks was positively correlated with baseline HbA1c, hsCRP, and systolic blood pressure. *How might your results change the direction of research or the focus of clinical practice?*: (i) There is a correlation between weight loss efficacy of exenatide and obesity. (ii) Overweight or obese T2DM patients can respond better to exenatide without an increased risk of excess weight loss. (iii) Weight loss efficacy of exenatide is also influenced by hyperglycemia. Compared to those with normal blood glucose levels, patients with poor blood glucose control can benefit from the weight loss effect of exenatide. (iv) The presence of atherosclerosis did not affect the weight loss benefit of exenatide.

## Supplementary Materials

Supplementary Materials Supplemental Figure 1. Comparison of BMI change in the normal weight group (BMI <24 kg/m^2^) and the overweight and obesity group (BMI ≥24 kg/m^2^) after exenatide treatment for 52 weeks. Supplemental Figure 2. Comparison of BMI change in the HbA1c <9% group and the HbA1c ≥ 9% group after exenatide treatment for 52 weeks. Supplemental Figure 3. Comparison of BMI change in the mild atherosclerotic group (cIMT <1 mm) and severe atherosclerotic group (cIMT ≥1 mm) after exenatide treatment for 52 weeks. Additional file 1: Flow diagram. Additional file 2: STROBE-checklist cohort.
