# Supplementary figures and images for: Efficacy of Exenatide Administered Twice Daily in Body Mass Index Reduction in Patients with Type 2 Diabetes Mellitus
Source: Int J Clin Pract. 2022 Oct 10;2022:7128859. doi: 10.1155/2022/7128859 (PMC10199797; doi:10.1155/2022/7128859)

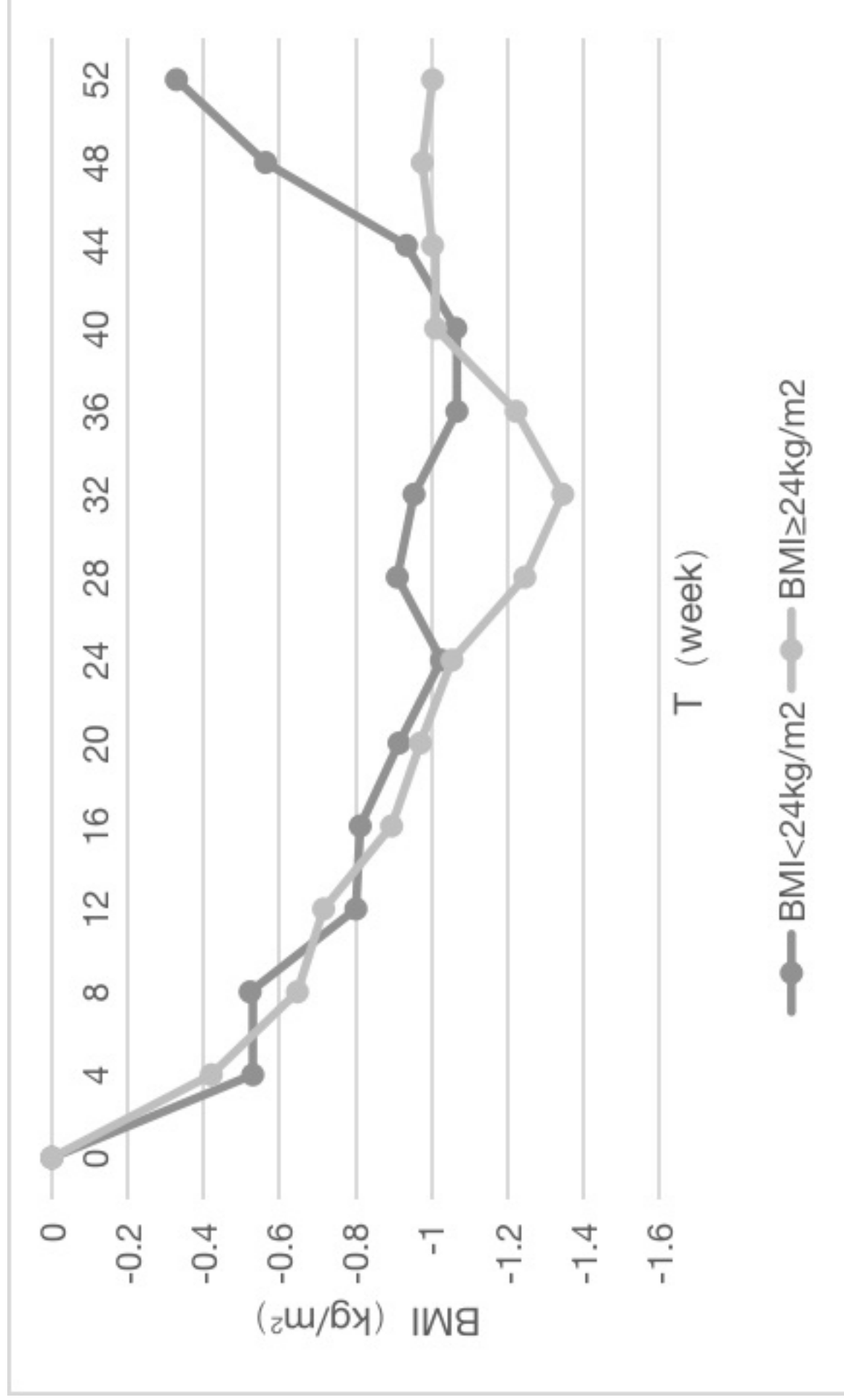

Supplement: Supplementary Materials — Supplemental Figure 1. Comparison of BMI change in the normal weight group (BMI <24 kg/m2) and the overweight and obesity group (BMI ≥24 kg/m2) after exenatide treatment for 52 weeks. Supplemental Figure 2. Comparison of BMI change in the HbA1c <9% group and the HbA1c ≥ 9% group after exenatide treatment for 52 weeks. Supplemental Figure 3. Comparison of BMI change in the mild atherosclerotic group (cIMT <1 mm) and severe atherosclerotic group (cIMT ≥1 mm) after exenatide treatment for 52 weeks. Additional file 1: Flow diagram. Additional file 2: STROBE-checklist cohort. [file 7128859.f1.zip › Sup_Fig.1.pdf]

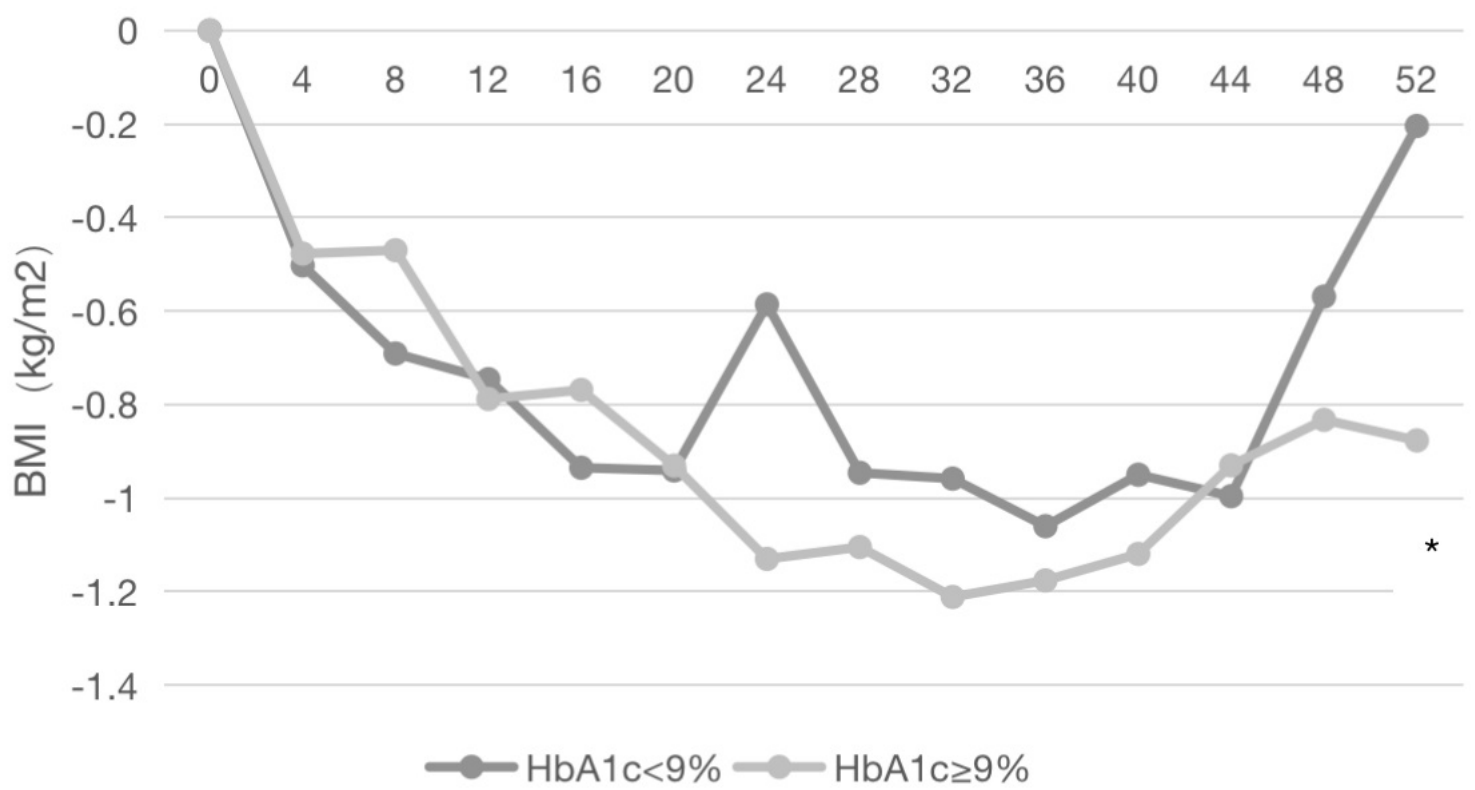

Supplement: Supplementary Materials — Supplemental Figure 1. Comparison of BMI change in the normal weight group (BMI <24 kg/m2) and the overweight and obesity group (BMI ≥24 kg/m2) after exenatide treatment for 52 weeks. Supplemental Figure 2. Comparison of BMI change in the HbA1c <9% group and the HbA1c ≥ 9% group after exenatide treatment for 52 weeks. Supplemental Figure 3. Comparison of BMI change in the mild atherosclerotic group (cIMT <1 mm) and severe atherosclerotic group (cIMT ≥1 mm) after exenatide treatment for 52 weeks. Additional file 1: Flow diagram. Additional file 2: STROBE-checklist cohort. [file 7128859.f1.zip › Sup_Fig.2.pdf]

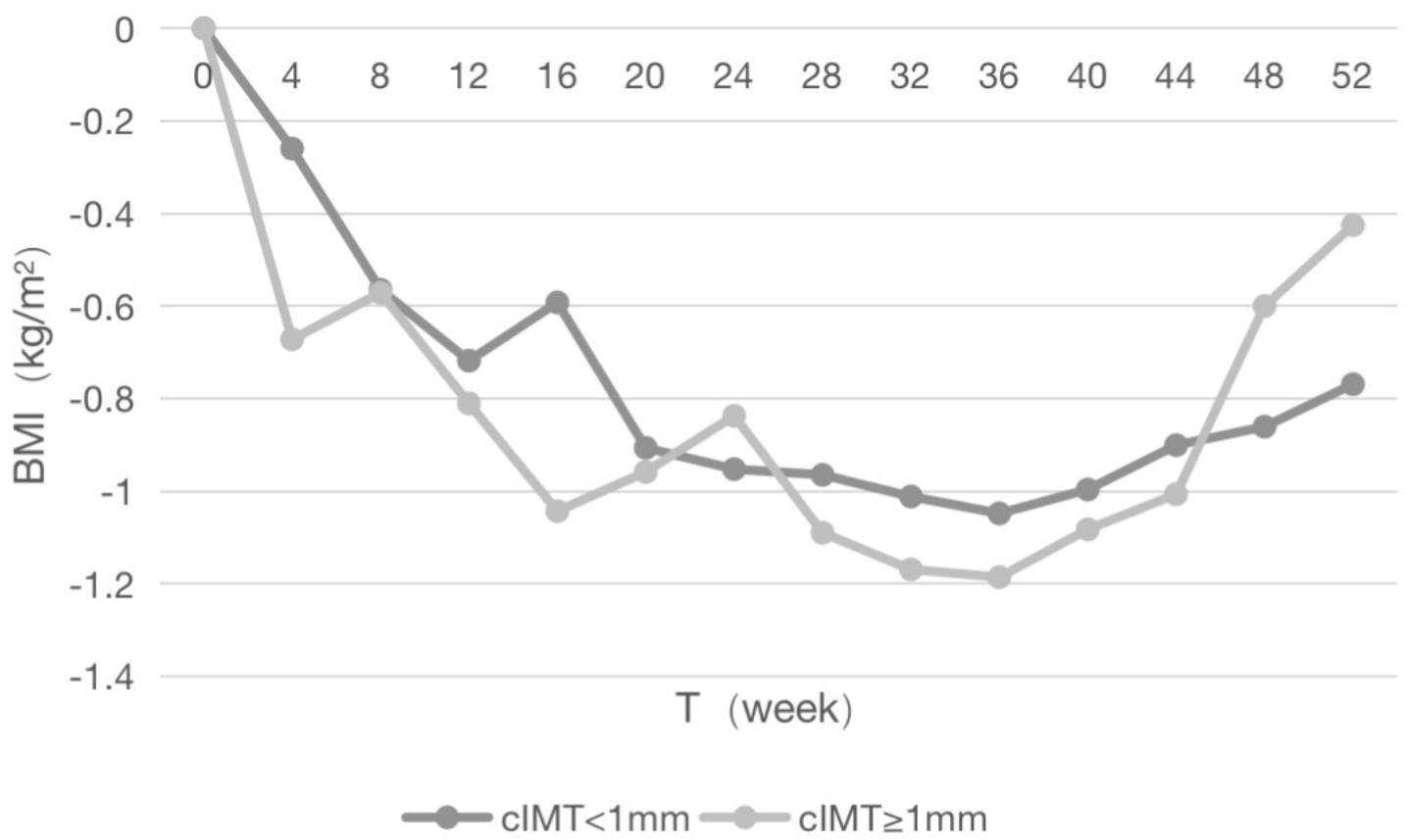

Supplement: Supplementary Materials — Supplemental Figure 1. Comparison of BMI change in the normal weight group (BMI <24 kg/m2) and the overweight and obesity group (BMI ≥24 kg/m2) after exenatide treatment for 52 weeks. Supplemental Figure 2. Comparison of BMI change in the HbA1c <9% group and the HbA1c ≥ 9% group after exenatide treatment for 52 weeks. Supplemental Figure 3. Comparison of BMI change in the mild atherosclerotic group (cIMT <1 mm) and severe atherosclerotic group (cIMT ≥1 mm) after exenatide treatment for 52 weeks. Additional file 1: Flow diagram. Additional file 2: STROBE-checklist cohort. [file 7128859.f1.zip › Sup_Fig.3.pdf]
