# Supplementary material for: Efficacy of Exenatide Administered Twice Daily in Body Mass Index Reduction in Patients with Type 2 Diabetes Mellitus
Source: Int J Clin Pract. 2022 Oct 10;2022:7128859. doi: 10.1155/2022/7128859 (PMC10199797; doi:10.1155/2022/7128859)
Supplement: Supplementary Materials — Supplemental Figure 1. Comparison of BMI change in the normal weight group (BMI <24 kg/m2) and the overweight and obesity group (BMI ≥24 kg/m2) after exenatide treatment for 52 weeks. Supplemental Figure 2. Comparison of BMI change in the HbA1c <9% group and the HbA1c ≥ 9% group after exenatide treatment for 52 weeks. Supplemental Figure 3. Comparison of BMI change in the mild atherosclerotic group (cIMT <1 mm) and severe atherosclerotic group (cIMT ≥1 mm) after exenatide treatment for 52 weeks. Additional file 1: Flow diagram. Additional file 2: STROBE-checklist cohort. [file 7128859.f1.zip › Additional file 1 Flow-Diagram.doc]

**CONSORT 2010 Flow Diagram**

**Allocation**

**Analysis**

**Follow-Up**

**Enrollment**

Assessed for eligibility (n=80 )

Excluded (n= 14 )

  Not meeting inclusion criteria (n=14 )

Analysed (n= 27 )
 Excluded from analysis (give reasons) (n=0 )

Lost to follow-up (1 withdraw with rash,3 withdraw for the blood glucose did not improve significantly after the maximum dosage,2loss of connection) (n= 6 )

Allocated to intervention (n= 33 )

 Received allocated intervention (n=33 )

Lost to follow-up (loss connection) (n=1 )

Allocated to intervention (n=33 )

 Received allocated intervention (n=33 )

Randomized (n=66 )
